# Laparoscopic Management of Spontaneous Heterotopic Pregnancy With Preservation of Intrauterine Pregnancy

**DOI:** 10.7759/cureus.107190

**Published:** 2026-04-16

**Authors:** Maria Sonia Rodriguez, Christopher Kaleb Romero Ríos, Geraldiny Mayorga, Catherine Scarlett Moreno Cabrera, Lorenzo E Aragón Conrado, Francgiliz J Robleto

**Affiliations:** 1 Obstetrics and Gynaecology, Hospital Militar Escuela "Dr. Alejandro Dávila Bolaños", Managua, NIC; 2 School of Medicine, Hospital Militar Escuela "Dr. Alejandro Dávila Bolaños", Managua, NIC; 3 Medical Education, Escuela de Medicina Teniente Coronel y Doctor Sergio Martinez Ordoñez, Managua, NIC; 4 Medical Education, Hospital Militar Escuela "Dr. Alejandro Dávila Bolaños", Managua, NIC; 5 Internal Medicine, Hospital Militar Escuela "Dr. Alejandro Dávila Bolaños", Managua, NIC

**Keywords:** diagnostic laparoscopy, salpingectomy, spontaneous heterotopic pregnancy, threatened abortion, viable intrauterine pregnancy

## Abstract

Heterotopic pregnancy, defined as the coexistence of an intrauterine and an ectopic pregnancy, is a rare condition in spontaneous conceptions and may delay diagnosis. We report the case of a 33-year-old Rh-negative, non-alloimmunized woman with a history of cesarean section who presented with metrorrhagia and pelvic pain at 7+1 weeks of pregnancy. Initial transvaginal ultrasound showed a viable intrauterine pregnancy with a subchorionic hematoma and a FIGO type 5 fibroid, without evidence of ectopic pregnancy. During hospital follow-up for threatened miscarriage, persistent symptoms prompted a repeat ultrasound, revealing a right adnexal structure consistent with a second gestational sac with cardiac activity. Diagnostic laparoscopy confirmed a right tubal ectopic pregnancy, and laparoscopic salpingectomy was performed with preservation of the intrauterine pregnancy. The postoperative course was uneventful, and the patient achieved delivery at term by cesarean section. This case highlights the diagnostic challenge of heterotopic pregnancy, which may be missed despite confirmation of an intrauterine pregnancy, and underscores the importance of early clinical suspicion and timely intervention.

## Introduction

Heterotopic pregnancy, defined as the coexistence of an intrauterine and an ectopic pregnancy, has a low incidence in spontaneous conceptions (1 in 30,000), although this increases significantly with assisted reproductive technologies (ART) [1,2]. Heterotopic pregnancy may be overlooked despite confirmation of an intrauterine pregnancy, leading to false reassurance and delayed diagnosis [3]. This delay increases the risk of tubal rupture and intra-abdominal hemorrhage, which may result in significant maternal morbidity [4]. Therefore, heterotopic pregnancy should always be considered in the differential diagnosis of patients presenting with persistent abdominal pain or vaginal bleeding in early pregnancy. In resource-limited settings such as Nicaragua, where access to advanced diagnostic tools may be restricted, clinical suspicion plays a critical role. To our knowledge, this represents one of the first reported cases in the country of spontaneous heterotopic pregnancy successfully managed by laparoscopy with preservation of the intrauterine pregnancy and favorable maternal and fetal outcomes.

## Case presentation

We present the case of a 33-year-old woman. She had a history of a prior low transverse cesarean section and was Rh-negative, non-alloimmunized. She presented to the obstetric emergency department with a 24-hour history of transvaginal bleeding and colicky pelvic pain, rated 7/10 on the visual analog scale, at 7+1 weeks of pregnancy, previously confirmed by last menstrual period and pregnancy testing.

Vital signs on admission were within normal limits. Gynecological examination revealed no active bleeding, although scant hematic residues were observed in the vaginal canal.

A transvaginal ultrasound performed at 7+1 weeks showed a viable intrauterine pregnancy with a gestational sac of appropriate size, a normal yolk sac, and a single embryo with cardiac activity (fetal heart rate: 134 bpm). A subchorionic hematoma measuring approximately 25 × 10 mm was identified adjacent to the inferior pole of the gestational sac. Additionally, a single anterior intramural uterine myoma measuring approximately 45 x 10 mm was observed, without apparent impact on the diagnosis or management.

Initial laboratory evaluation, including complete blood count, renal and liver function tests, and urinalysis, was within normal limits.

Based on the clinical and sonographic findings, a diagnosis of threatened miscarriage was made. Conservative management was initiated with vaginal micronized progesterone (200 mg every 12 hours), relative rest, and hospital surveillance, according to institutional practice. Short-term indomethacin was administered and subsequently discontinued.

During a 78-hour hospital stay, the patient continued to experience moderate transvaginal bleeding and intermittent pelvic pain.

**Figure 1:**
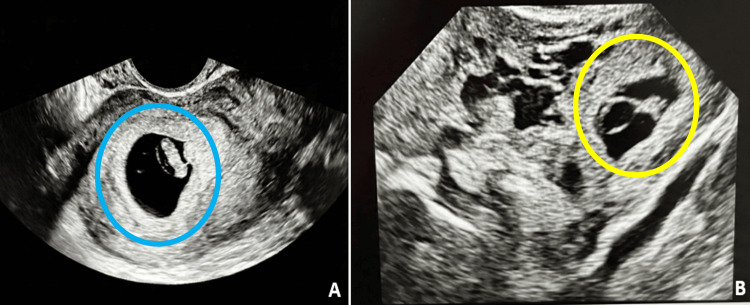
Obstetric ultrasound at 7 + 4 weeks of pregnancy. A. The blue circle shows a yolk sac at the intrauterine level. B. The yellow circle demonstrates the yolk sac in the right adnexa measuring 24 x 18 mm.

A follow-up transvaginal ultrasound showed persistence of the viable intrauterine pregnancy with cardiac activity (fetal heart rate: 105 bpm). Additionally, a complex right adnexal structure measuring 24 × 18 mm was identified, containing a gestational sac with an embryonic pole and cardiac activity, raising suspicion of heterotopic pregnancy (Figure 1).

A diagnostic laparoscopy was performed, confirming a right ampullary ectopic pregnancy with a visible gestational sac and mild peritubal hemorrhage. A laparoscopic right salpingectomy was performed without complications, with an estimated blood loss of less than 100 mL. Anti-D immunoglobulin (300 µg IM) was administered intraoperatively due to the patient’s Rh-negative status.

In the immediate postoperative period, a control ultrasound confirmed persistence of the viable intrauterine pregnancy with preserved cardiac activity. At 30 hours post-procedure, laboratory tests showed hemoglobin and hematocrit within normal limits. Leukocytosis (15,400/µL) with neutrophilia (78%) and elevated C-reactive protein (22 mg/L) were observed, with negative procalcitonin (<0.05 ng/mL), consistent with a postoperative inflammatory response in the absence of infection. Laboratory values are shown in Table 1.

**Table 1:** Laboratory values

Laboratory Test	Patient Result	Reference Range
Hemoglobin	13.4 g/dL	12.0 – 16.0 g/dL
Hematocrit	40.1 %	36.0 – 46.0 %
Mean Corpuscular Volume (MCV)	88.0 fL	80.0 – 100.0 fL
Platelets	265,000 /µL	150,000 – 450,000 /µL
White Blood Cells (WBC)	15,400 /µL	4,500 – 11,000 /µL
Neutrophils	78 %	40 – 70 %
Lymphocytes	16 %	20 – 40 %
C-Reactive Protein (CRP)	22 mg/L	< 5.0 mg/L
Procalcitonin	0.03 ng/mL	< 0.05 ng/mL

The patient had a favorable clinical course, with resolution of vaginal bleeding and progressive normalization of inflammatory parameters. She was discharged with recommendations for relative rest, sexual abstinence, and weekly ultrasound follow-up. At a one-week outpatient follow-up, ultrasound confirmed a viable intrauterine pregnancy without evidence of an active hematoma.

The pregnancy progressed without complications. At 38 weeks of pregnancy, an elective cesarean section was performed due to prior uterine surgery, resulting in the delivery of a healthy female neonate appropriate for gestational age, with Apgar scores of 8 and 9 at 1 and 5 minutes, respectively.

Over the following days, the patient evolved favorably, with resolution of transvaginal bleeding and progressive decrease of inflammatory parameters. She was discharged with instructions for relative rest, sexual abstinence, and weekly ultrasound follow-up. Ambulatory control one week later showed a viable intrauterine pregnancy with no active hematoma.

**Figure 2:**
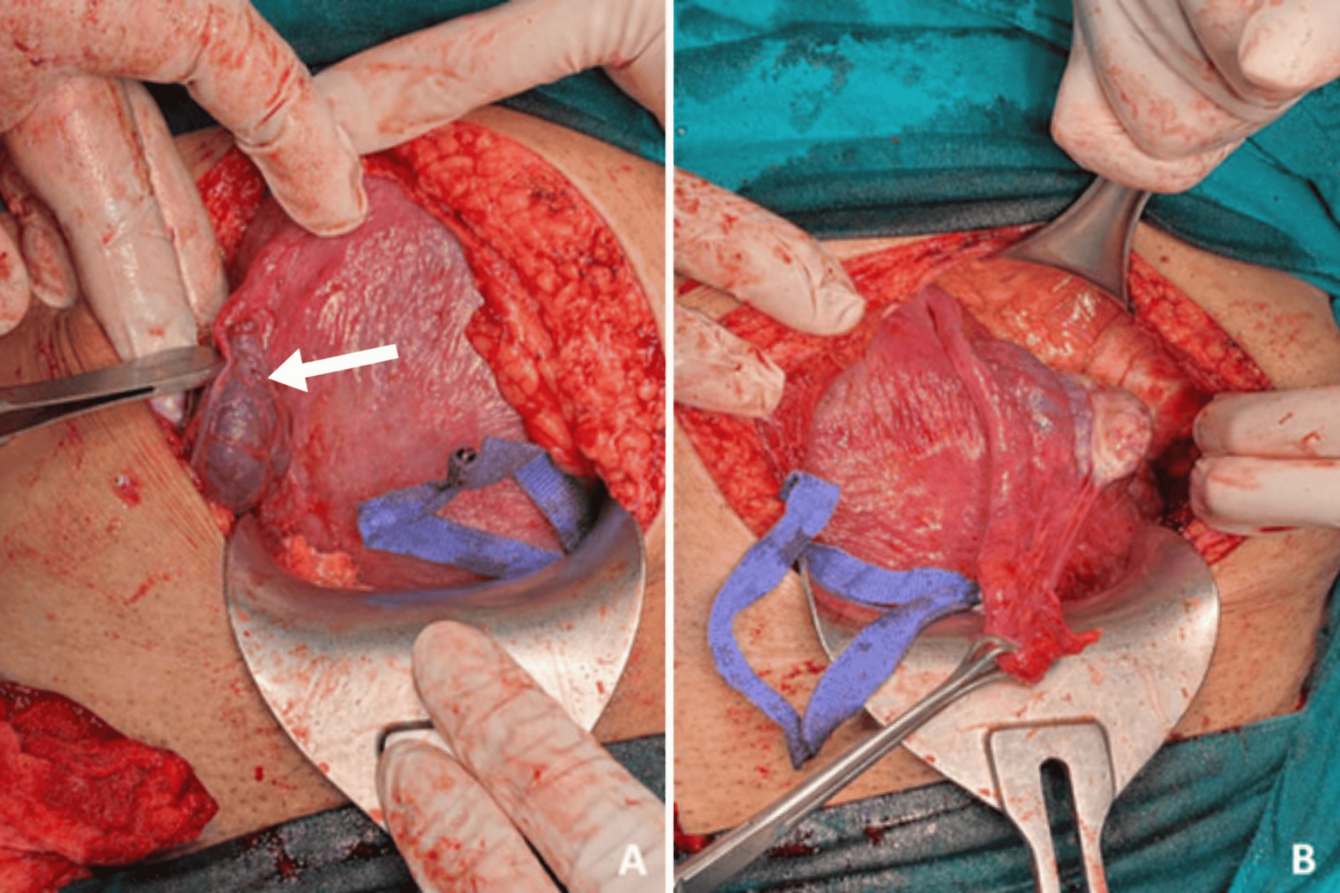
Intraoperative findings during elective cesarean section at term. A: Linear surgical scar in the right Fallopian tube, corresponding to the site of previous ectopic implantation and prior salpingectomy. B: Left adnexa appearing normal.

During the procedure, a linear surgical scar was observed in the right Fallopian tube, corresponding to the previous site of ectopic implantation and salpingectomy. The left adnexa appeared normal (Figure 2). The postoperative course was uneventful, and both mother and newborn were discharged in good clinical condition.

## Discussion

Heterotopic pregnancy, defined as the coexistence of an intrauterine gestation with an ectopic one, represents a diagnostic and therapeutic challenge, especially in the current context of increased use of assisted reproductive technologies (ART). In spontaneous pregnancies, the incidence is extremely low, estimated at approximately 1 per 30,000 gestations [1]. However, this risk increases substantially in women undergoing ART, reaching rates of 1 in 100 to 1 in 500 clinical pregnancies following in vitro fertilization (IVF) [1-3]. Most currently documented cases are related to the use of these technologies, with no relevant differences between cycles using fresh or frozen embryos [3]. Although epidemiological data in Central America are limited, trends are considered to follow the same global pattern with a progressive increase in cases associated with the growing use of ART.

Heterotopic pregnancy can trigger potentially lethal complications, mainly due to intra-abdominal hemorrhage secondary to the rupture of the ectopic pregnancy, a condition that presents acutely in about 50% of cases [4]. Despite the maternal risk, fetal survival of the concomitant intrauterine pregnancy can exceed 65-75% when adequate and timely management is provided [5,6].

Various risk factors have been associated with the development of heterotopic pregnancy. Among the most important are the use of IVF and multiple embryo transfers [1-3], a history of pelvic inflammatory disease (PID) [1,3,6], previous tubal surgeries or salpingectomies [1,3], a history of ectopic pregnancy [3], uterine or tubal malformations, and conditions involving ovarian stimulation, including ovarian hyperstimulation syndrome (OHSS) [1].

From a pathophysiological standpoint, this type of pregnancy occurs when, following multiple ovulations or the transfer of more than one embryo, one or more embryos implant outside the endometrial cavity while another embryo implants correctly within the uterus. In the context of ART, factors such as embryo manipulation and previous tubal damage may favor embryonic migration towards extrauterine locations [1-3].

The most frequent clinical manifestations include abdominal pain (in 60-70% of cases) and vaginal bleeding (50-60%), although up to 20% may be asymptomatic at initial evaluation [7]. Therefore, transvaginal ultrasound should be used systematically in patients with risk factors, especially after fertility treatments [7].

In this patient, suspicion arose following a suggestive sonographic finding in the right adnexa during hospital follow-up for threatened abortion, which prompted a diagnostic laparoscopy. This approach allowed for the confirmation of the ectopic pregnancy in the right tube and the performance of a salpingectomy, with preservation of the intrauterine pregnancy. The most common ectopic location in these cases is the Fallopian tube, as occurred here, although it can also be located in other extrauterine areas [1-3].

The management of heterotopic pregnancy must be individualized, considering both the viability of the intrauterine pregnancy and maternal safety, as was done in this case [7]. Available therapeutic options include conservative surgical interventions and minimally invasive techniques; laparoscopy is preferred due to its lower morbidity and better preservation of the intrauterine pregnancy. However, in situations of hemodynamic instability or massive hemoperitoneum, laparotomy is chosen. Additionally, in highly selected cases, local agents such as potassium chloride (KCl) or hyperosmolar glucose may be used to resolve the ectopic gestation without compromising the intrauterine pregnancy [8].

## Conclusions

This case highlights the diagnostic challenge of heterotopic pregnancy, particularly in spontaneous conceptions where clinical suspicion may be low. Persistent abdominal pain or bleeding should prompt reassessment, even in the presence of a confirmed intrauterine pregnancy. Timely diagnosis and minimally invasive surgical management allowed preservation of the intrauterine pregnancy and favorable maternal and fetal outcomes. Strengthening clinical awareness and promoting systematic ultrasound evaluation are essential, especially in resource-limited settings.

Clinicians should recognize that the presence of an intrauterine pregnancy does not exclude a concurrent ectopic pregnancy, and persistent symptoms must prompt further evaluation.
